# Soil fungal community structure and function response to rhizoma perennial peanut cultivars

**DOI:** 10.1186/s12870-024-05209-y

**Published:** 2024-06-19

**Authors:** Umar Daraz, Adesuwa S. Erhunmwunse, José C. B. Dubeux, Cheryl Mackowiak, Hui-Ling Liao, Xiao-Bo Wang

**Affiliations:** 1 State Key Laboratory of Herbage Improvement and Grassland Agro-Ecosystems, College of Pastoral, Agriculture Science and Technology, Center for Grassland Microbiome, Lanzhou University, Lanzhou, China; 2 North Florida Research and Education Center, University of Florida, Quincy, FL USA; 3 North Florida Research and Education Center, University of Florida, Marianna, FL USA

**Keywords:** Legumes, Soil fungi, Microbial diversity, Plant-microorganism interactions, Grassland ecosystem

## Abstract

**Background:**

Crop-associated microorganisms play a crucial role in soil nutrient cycling, and crop growth, and health. Fine-scale patterns in soil microbial community diversity and composition are commonly regulated by plant species or genotype. Despite extensive reports in different crop or its cultivar effects on the microbial community, it is uncertain how rhizoma peanut (RP, *Arachis glabrata* Benth.), a perennial warm-season legume forage that is well-adapted in the southern USA, affects soil microbial community across different cultivars.

**Results:**

This study explored the influence of seven different RP cultivars on the taxonomic composition, diversity, and functional groups of soil fungal communities through a field trial in Marianna, Florida, Southern USA, using next-generation sequencing technique. Our results showed that the taxonomic diversity and composition of the fungal community differed significantly across RP cultivars. Alpha diversity (Shannon, Simpson, and Pielou’s evenness) was significantly higher in Ecoturf but lower in UF_Peace and Florigraze compared to other cultivars (*p* < 0.001). Phylogenetic diversity (Faith’s PD) was lowest in Latitude compared to other cultivars (*p* < 0.0001). The dominant phyla were Ascomycota (13.34%), Mortierellomycota (3.82%), and Basidiomycota (2.99%), which were significantly greater in Florigraze, UF_Peace, and Ecoturf, respectively. The relative abundance of *Neocosmospora* was markedly high (21.45%) in UF_Tito and showed large variations across cultivars. The relative abundance of the dominant genera was significantly greater in Arbrook than in other cultivars. There were also significant differences in the co-occurrence network, showing different keystone taxa and more positive correlations than the negative correlations across cultivars. FUNGuild analysis showed that the relative abundance of functional guilds including pathogenic, saprotrophic, endophytic, mycorrhizal and parasitic fungi significantly differed among cultivars. Ecoturf had the greatest relative abundance of mycorrhizal fungal group (5.10 ± 0.44), whereas UF_Peace had the greatest relative abundance of endophytic (4.52 ± 0.56) and parasitic fungi (1.67 ± 0.30) compared to other cultivars.

**Conclusions:**

Our findings provide evidence of crop cultivar’s effect in shaping fine-scale fungal community patterns in legume-based forage systems.

**Graphical abstract:**

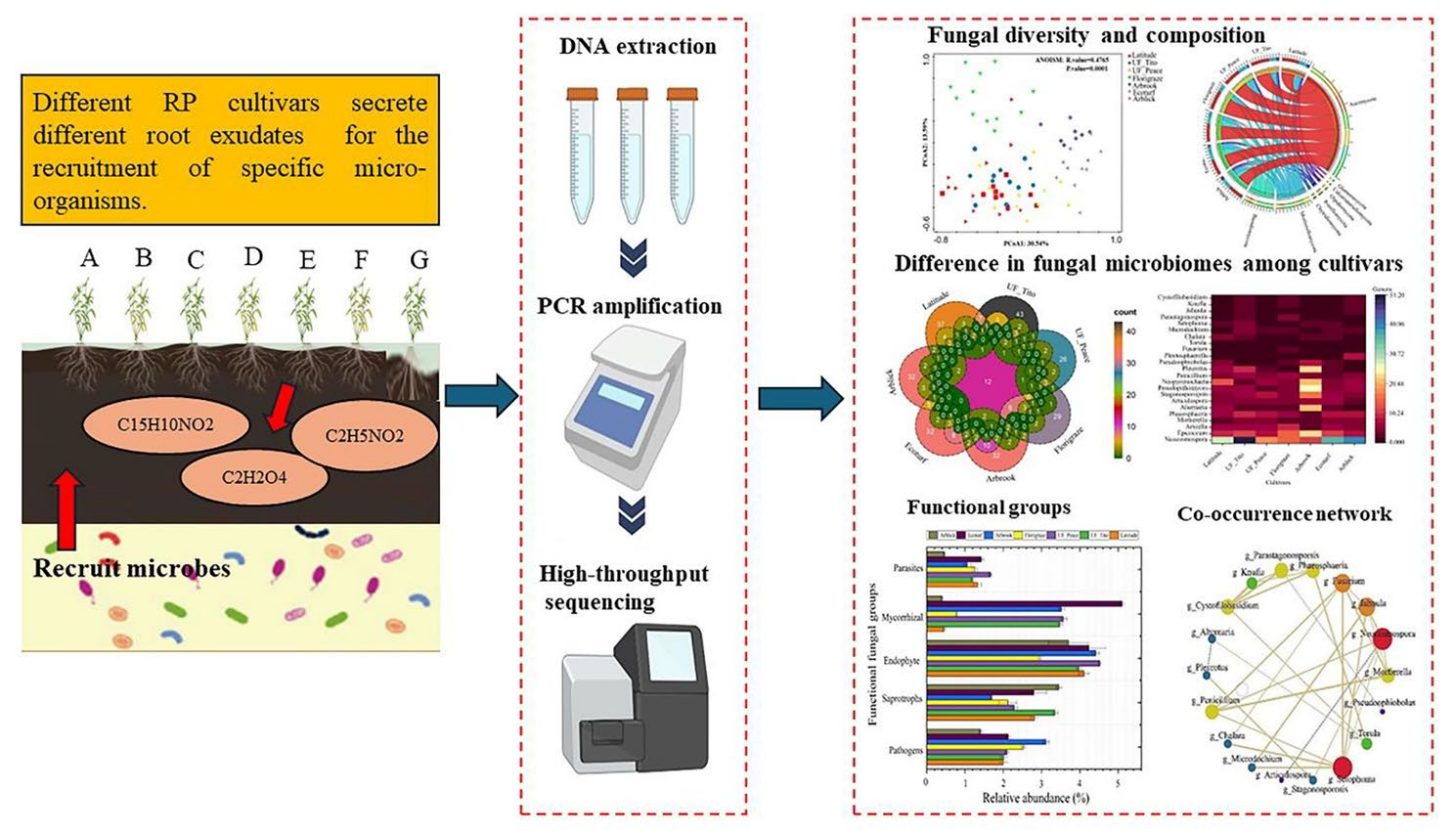

**Supplementary Information:**

The online version contains supplementary material available at 10.1186/s12870-024-05209-y.

## Background

In agricultural ecosystems, there is an increasing demand to boost food production in response to the rapid growth of the global population. However, the traditional approach that relies on high external inputs to increase crop yield has been demonstrated to be unsustainable in agricultural management practices [1]. Crop-associated microbiomes play an important role in regulating many key ecological processes, including carbon (C) and nitrogen (N) cycling [2], nutrient acquisition [3], and soil formation [4], consequently affecting crop growth and health [5]. In a recent study, early season soil microbiome has been shown a better ability for predicting wheat grain quality [6]. Soil fungi, such as *Penicillium* [7], *Trichoderma* [8], and arbuscular mycorrhiza fungus (AMF) [9], can promote plant growth. These fungal members, known as plant growth promoting fungi (PGPF), exert a positive effect on plants by a variety of mechanisms, such as facilitating plant nutrient uptake (e.g., nitrogen and phosphorous) [2], and enhancing plant resistance against abiotic stresses [10] and pathogenic microorganisms [11]. Understanding fungal diversity, composition and functions, and their relationships with crops is particularly important for the development of microbial-based indicators and fertilizers for sustainable agriculture and soil health [12, 13].

Soil fungi are highly diversified and serve crucial roles in ecosystems. Most of them are saprotrophs and can work as decomposers to promote nutrient cycling [14]. For example, Basidiomycota plays important roles in the decomposition of plant litter and complex organic materials (cellulose, lignin, and pectin) in soils [15], by producing extracellular hydrolytic and oxidative enzymes [16]. Some fungi can also form mutualistic relationships with their hosts [17, 18], which provide various benefits to plant growth including nutrient mobilization, hormone production, biological dinitrogen fixation (BNF), and drought resistance [19, 20]. For example, mutualistic, endophytic fungus *Piriformospora indica* has been shown to improve plant growth, increase nutrient uptake, and enhance resistance to various stresses such as drought and salinity [21]. Numerous reports have shown that AMF are one of the most important groups of symbionts that live among the roots of most plant species. These fungi enhance plant growth by promoting root formation [22] and optimizing resistance to abiotic stresses [23]. Previous studies have reported that soil fungi have biological control actions against pathogenic microorganisms, which generate a favorable impact on plant productivity [24, 25].

Soil microbial communities are generally influenced by climatic and soil factors [26, 27], but fine-scale assembly patterns in microbial communities are closely associated with host plant characteristics such as root exudates and root morphology. Due to the differences in root structure and exudates [28], volatile organic compounds [29], and quality and quantity of carbon input [30], different plant species or genotype can serve to induce specific taxa of microbial communities in their rhizosphere and recruit symbiotic organisms to roots. In crop systems, growing evidence has shown that the composition and diversity of soil microbial communities varied largely among cultivars in various crops [31, 32], including wheat [33], rice [34], sorghum [35], chickpea [36] and peanut [37, 38]. Understanding the effects of different plant species or genotypes on microbial communities is important for optimizing agricultural practices, enhancing soil health, and promoting sustainable crop production through modulating plant-microorganism interactions. However, our understanding of cultivar effects on soil fungal communities remains very limited, especially in forage systems.

Rhizoma peanut (RP; *Arachis glabrata* Benth.) is a perennial warm-season legume forage that is adapted to sandy soils in southern USA [39, 40] and is a beneficial option for pasture integration [41]. RP has a considerable potential to increase soil N supply in grasslands. Like many other perennial legume species, RP supplies N through biological N_2_ fixation (BNF), which is a major source of N in agricultural systems (50–70 Tg N annually) [42]. When grown with companion grasses, N from RP can be shared with pasture grasses by plant litter, root exudates, and grazing animals via animal excreta [40, 43]. In addition, RP increases soil N accumulation and enriches litter quality in C4 grass pastures [44].

Several RP cultivars exist in Florida and serve different purposes. Some cultivars, such as Arbrook, UF_Peace, and UF_Tito, are grown as forages, while others serve as groundcover, like Ecoturf. Others are known for being disease-resistant, such as UF_Tito and UF_Peace. According to Dubeux et al. [40], the potential of BNF in RP is cultivar-dependent and may be linked to variation in the diversity and composition of the rhizosphere microbial communities. Ecoturf and Florigraze are the two most commonly used RP cultivars in Florida. Erhunmwunse et al. [27] reported changes in soil fungal communities across the two cultivars. However, further evidence is needed to evaluate the effects of these RP cultivars and other cultivars on soil fungal communities. In this study, we hypothesized that the host plant cultivar would lead to significant changes in soil fungal community structure and function. The objectives of this study are to (i) examine the changes in soil fungal community diversity and composition across different RP cultivars; and (ii) identify the changes in functional groups and keystone taxa of fungal communities among the different RP cultivars. “Keystone taxa are species within an ecosystem that have a disproportionately large impact on the structure and function of that ecosystem”.

This study characterized the soil fungal community structure and function under seven perennial RP cultivars under the same soil type and conditions. Our study lays the framework for understanding the role of RP-based forage systems on soil fungal composition, function, and contributions to pasture growth and soil health.

## Materials and methods

### Study site and sampling

This study was conducted at the North Florida Research and Education Center (NFREC) in Marianna, Florida, Southeastern United States (30°52′N, 85°11′W). The soil at the experimental site was Red Bay fine sandy loam fine-loamy, thermic, and kaolinitic, Rhodic Kandiudults; USDA Soil Survey Staff [45]. The average annual rainfall in the experimental area was 1360 mm over the past 30 years, and the average altitude was 35 m a.s.l. The average temperature in 2020 was 20.0 °C (6.7 and 31.7 °C min/max, respectively). A total of seven different cultivars (Arblick, Latitude, UF_Tito, UF_Peace, Florigraze, Arbrook and Ecoturf) were planted in September 2010. The experiment was established in a randomized complete block design with four replicates for each cultivar. The size of each plot was 2 m × 3 m, and there was a 2-m alleyway between the plots. Planting materials were obtained from the NFREC. In April 2015, the herbicide [5-methyl-2-(4-methyl-5-oxo-4-pro-pan-2-yl-1 H-imidazol-2-yl) py-ridine-3-carbo-xylic acid] was used. In addition, 56 and 74 kg ha^− 1^ potassium and 29 and 10 kg ha^− 1^ phosphorus were applied to these plots in June 2014 and April 2015, respectively. A detailed description of the plot design and management practices was reported in a previous study [40].

### Soil sampling and DNA extraction

In April 2017, we randomly selected three soil cores (3 cm diameter × 10 cm depth) in each plot, resulting in twelve soil samples per cultivar to minimize the random effect of spatial differences. A total of 84 samples were thus collected from the experimental field. Roots affect the entire soil surface because of the horizontal growth pattern of RP, which occurs via rhizomes. Therefore, the soil samples we collected comprised both bulk and rhizosphere soils. The samples were immediately placed in an icebox after being sealed in a sterile plastic bag and transported to the laboratory within 2 h. The soil samples were sieved using a 2-mm sieve to remove roots, debris, and rocks. Subsequently, the samples were thoroughly mixed to achieve homogeneity and then preserved at -80 °C for DNA extraction. Total soil DNA was extracted according to the manufacturer’s instructions using the Qiagen’s DNeasy PowerSoil Kit (Qiagen Inc., CA, USA). The quality and quantity of the extracts were evaluated using a spectrophotometer (NanoDrop (ND-ONE-W), ThermoFisher Scientific, Waltham, MA, USA). The quantity and quality of DNA were determined by measuring the absorbance ratios (A260/A280 and A260/A230) using a NanoDrop TM One (NanoDrop Technologies Inc., ThermoScientific, USA). The absorbance ratios ranging from 1.8 to 2.2 are considered indicative of high-quality DNA extraction.

### Amplicon sequencing data analysis

Composition and diversity of soil fungal community was determined using a modified form of the three-step PCR method targeting fungal ITS1 region, as Chen et al. [46] described. In brief, fungal ITS1 genes were amplified using the primer pair ITS1F (5’ -CTTGGTCATTTAGAGGAAGTAA-3’) and ITS2R (5’-GCTGCGTTCTTCATC GATGC-3’) for ten PCR cycles (first-step PCR). In addition to the sequencing primer, six frameshifting primers were used in ten additional PCR cycles (2nd -step PCR). The frameshifting primers were made up of the primer pair used in the first step of PCR along with frameshifting nucleotides. This was done to increase diversity and reduce sequence bias in the initial bases [47]. We then used the third-step PCR to add error-tolerant barcodes through ten more PCR cycles. Prior to aggregation, we individually purified the third-step PCR products using bead cleaning (AMPure XP, Beckman Instruments, Brea, CA, USA). A spectrophotometer (NanoDrop™) was used to measure the amount and quantity of PCR products. Moreover, 1.7% (w/v) agarose gels were used to screen the PCR products to confirm their size and quantity. The Illumina (Illumina Inc., San Diego, CA, USA) Miseq Nano (v2 250 bp, 500 Mb sequencing capability) at the Duke Center for Genomic and Computational Biology (GCB, Durham, NC, USA) was used to pool and sequence the barcode PCR products.

Sequencing data were processed at the Chinese Academy of Sciences Research Center for Eco-Environmental Sciences, (http://mem.rcees.ac.cn:8080/root/) using an in-house pipeline developed on the Galaxy platform [48]. In summary, the forward and reverse threads of the same sequence were combined using FLASH v1.2.5 to create a single sequence with at least 30 bp of overlap and 0.25 mismatches [49]. The sequences were then quality trimmed using Btrim [50] with a Phred-score threshold of 30 over a 5-bp window size. Next, sequences were clustered using UPARSE at the 97% identity threshold to create operational taxonomic units (OTUs) [51]. OTUs that had only one read (singletons) were removed. Based on the clustering results, the final OTUs were produced. The UNITE ITS reference database provided a taxonomy annotation for representative sequences of OTUs [17]. Using the resampled OTU table available at Dryad (10.5061/Dryad 8080), we rarefied all samples to an equal sampling depth of 1800 sequences for subsequent community analysis.

### Statistical analysis

Four indices, including Shannon index, Simpson index, Pielou’s evenness, and phylogenetic diversity (Faith’s PD), were used to evaluate fungal alpha diversity in this study. Based on the phylogenetic tree and OTU table, Faith’s PD was calculated using the *pd* function from the picante package in R version 3.5.3.​​​​​​​ The graphs of alpha diversity and the Venn diagram were made using omics studios (https://www.omicstudio.cn/*).* Principal coordinate analysis (PCoA) were used to visualize dissimilarity between samples using CANOCO 5 software (version 4.5 for Windows; Ithaca, NY, USA) (Canoco5, 2012). The *anosim* function in the vegan package was used to test the effects of cultivars on the fungal community structure. We selected ten dominant phyla and 20 dominant genera (mean relative abundance > 1% across all samples) to examine the differences in composition among seven different cultivars. Canoco software (version4.5 for Windows; Ithaca, NY, USA) was used to perform Principal component analysis (PCA) to investigate taxonomic distributions at the genus level across cultivars. Circos 0.67-7 software was used to display the Circos graph to reveal the changes in fungal taxonomic interactions across seven different cultivars. The network analysis was carried out using the Wekemo Bioincloud (https://www.bioincloud.tech/) for the 20 dominant bacterial genera across RP cultivars, which was measured by Spearman rank correlation coefficients with *p*-value of 0.05. If the data does not meet the assumptions of normality of variance, the data was log-transformed or square-root transformed. Significant differences in alpha diversity metrics were tested using the ANOVA with Tukey’s HSD test. FUNGuild was used to predict the functions of the fungal communities in seven different RP soil samples. The fungal functional group (guild) was determined by FUNGuild v1.0 [52]. We used FUNGuild to analyze the high-throughput sequencing datasets from the treatments and put them into three trophic modes based on fungi feeding habits: symbiotroph, saprotroph, and pathotroph. Significant differences were tested in the relative abundance of taxonomic groups at all taxonomic levels (phylum, family and genus) using one-way ANOVA with Tukey’s HSD test. Test results with *p* < 0.05 were considered statistically significant.

## Results

### Alpha diversity

**Fig. 1:**
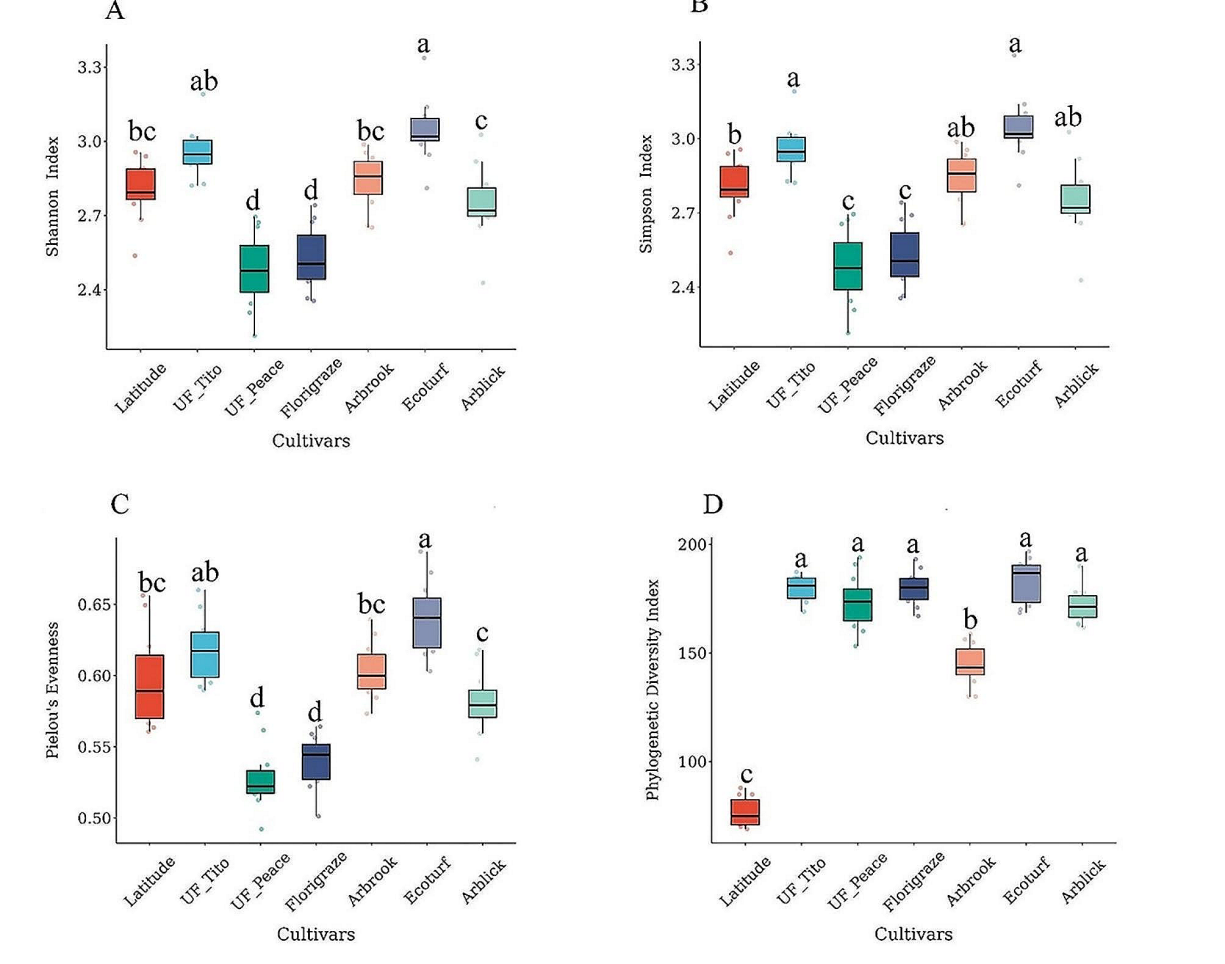
Shannon index (**A**), Simpson index (**B**) Pielou’s evenness index (**C**), and Phylogenetic diversity (**D**) of the fungal communities across seven rhizoma peanut cultivars in April. Colored dots represent individual data points. Different lowercase letters inicate significant differences (ANOVA, *p* < 0.05) among cultivars

A total of 622,361 high-quality sequences were obtained for soil fungi across all samples. After clustering sequences at the 97% similarity threshold and removing singletons, we obtained 1853 operational taxonomic units (OTUs) for fungi. Overall, alpha diversity significantly differed among cultivars (*p* < 0.01) (Fig. 1 and Table S1). Ecoturf had the greatest alpha diversity estimated using Shannon, Simpson, and Pielou’s evenness indices, whereas UF_Peace and Florigraze had the least alpha diversity than other cultivars (*p* < 0.001) (Fig. 1A, B, C and Table S1). Latitude had the lowest alpha diversity estimated by Faith’s PD compared to other cultivars (*p* < 0.0001) (Fig. 1D and Table S1).

**Fig. 2:**
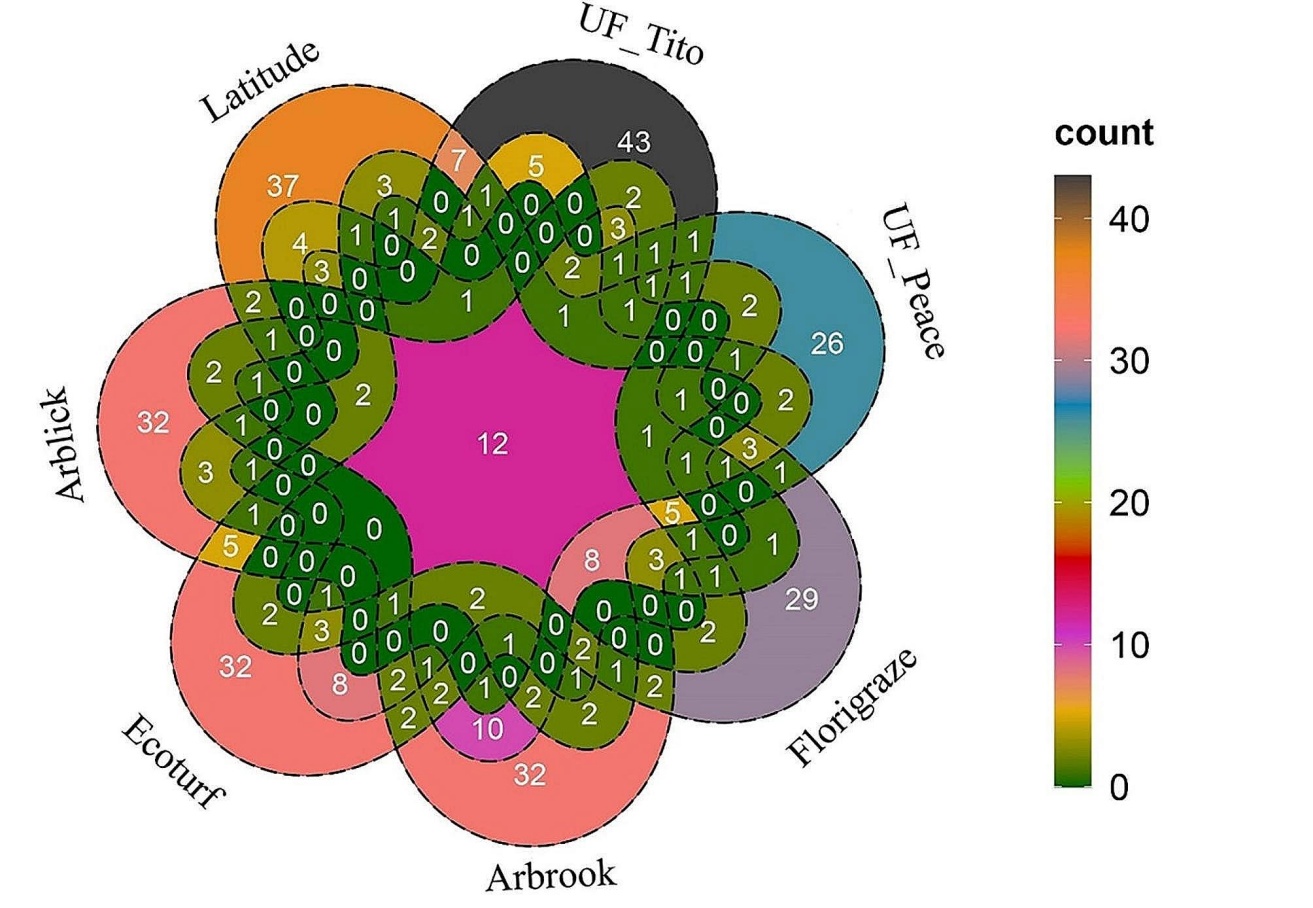
Venn diagram of the number of operational taxonomic units (OTUs) of fungi obtained across seven different RP cultivars

Comparison of the fungal OTUs shared among the seven different RP cultivars showed that the number of unique OTUs in Latitude, UF_Tito, UF_Peace, Florigraze, Arbrook, Ecoturf, and Arblick cultivars were 486, 477, 498, 472, 461, 489, and 500, respectively. The shared fungal OTUs among the seven RP cultivars were 117, which made up 24.21% of the total OTUs (Fig. 2).

### Beta diversity and fungal community composition

**Fig. 3:**
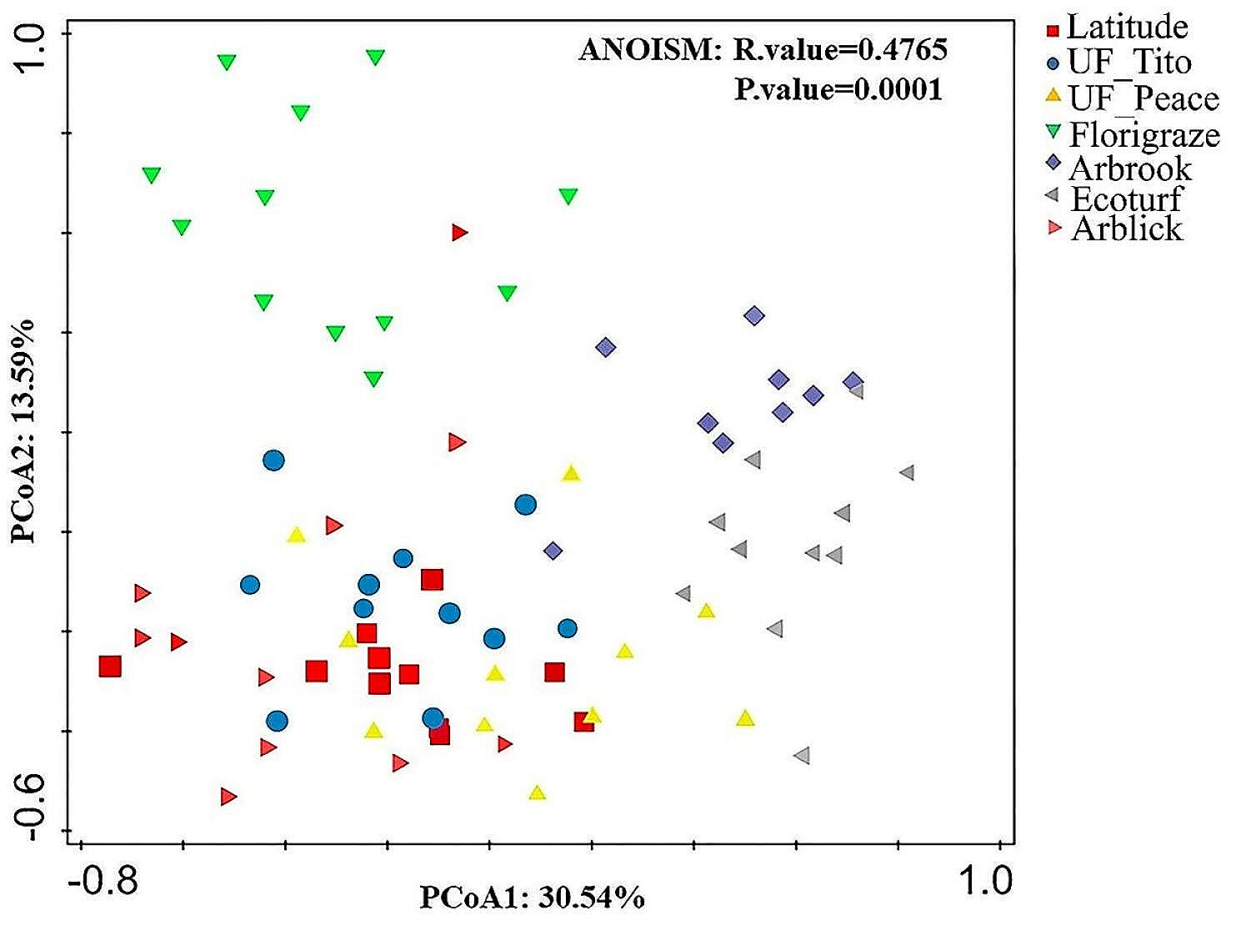
Principal coordinate analysis (PCoA) of fungal β diversity across rhizoma peanut cultivars based on Bray-Curtis distance matrices

Variations in fungal community structures among RP cultivars were shown on the first two axes of the PCoA (Fig. 3). The first and second axis explained 30.54% and 13.59% of the variance in fungal communities, respectively (Fig. 3). Fungal community structure significantly differed across seven cultivars (*R* = 0.48; *p =* 0.0001).

**Fig. 4:**
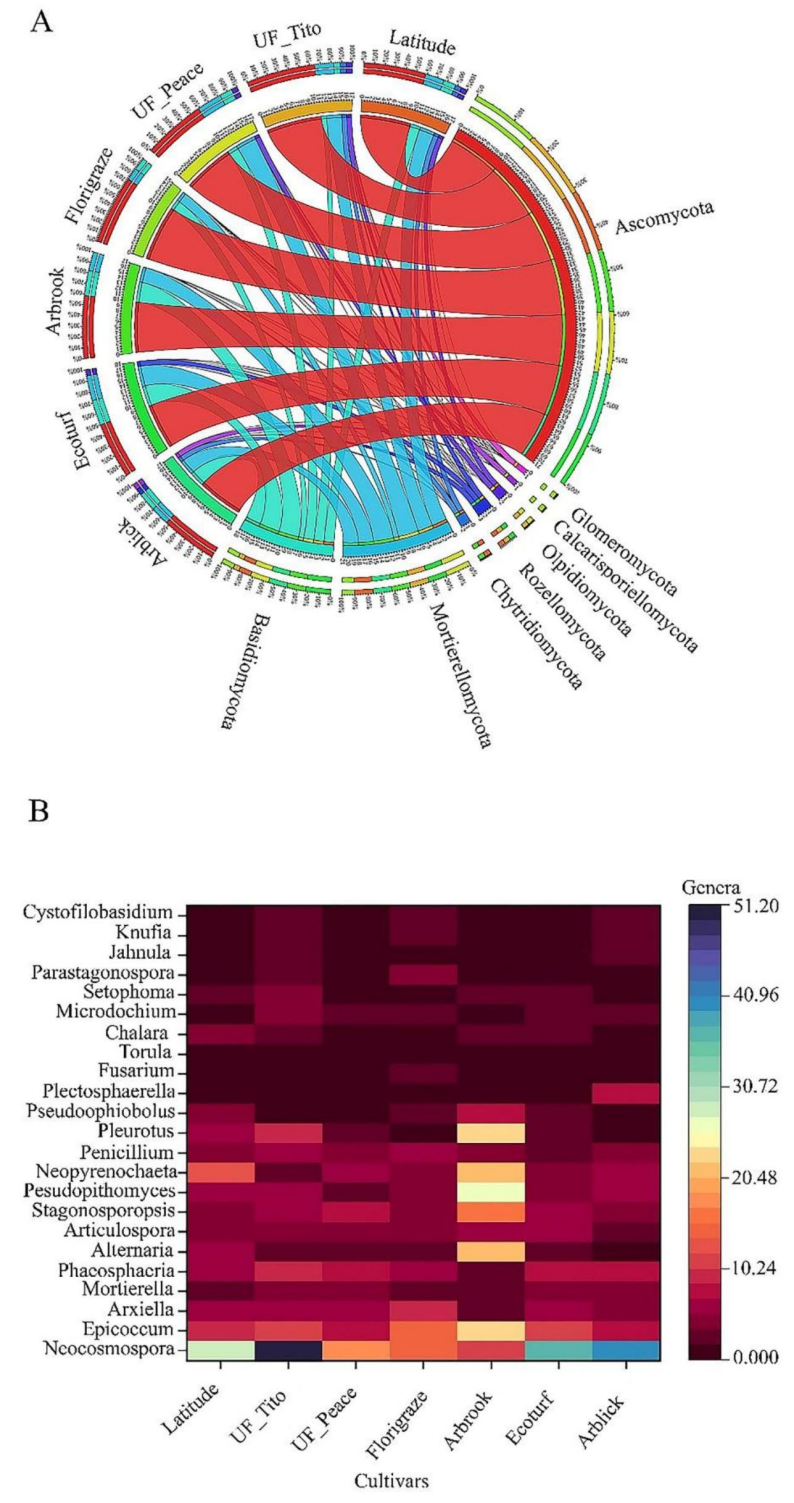
Relative abundance of the dominant fungal groups at the phylum level (**A**) and at the genera level fungi (**B**) across rhizoma peanut cultivars. The data was visualized via Circos software (http://circos.ca/*).* The thickness of each ribbon in represented the relative abundance of taxa in each phyla group. In the taxonomic OTU bubble plot, circle sizes represent the relative abundance of the group at the family level. In the heat map of genera, the color bar indicates the range of contribution of a genus across cultivars

At the phylum level, a total of eight dominated phyla of fungi were present across all cultivars including, Ascomycota, Basidiomycota, Mortierellomycota, Chytridiomycota, Rozellomycota, Olpidiomycota, Calcarisporiellomycota, Glomeromycota, Mucoromycota, Blastocladiomycota, and Entomophthoromycota. Of these phyla, Ascomycota (13.34%), Mortierellomycota (3.82%), and Basidiomycota (2.99%) were the dominant taxa (Fig. 4A). The relative abundance of the dominant fungal taxa was significantly different among RP cultivars (*p* < 0.05) (Fig. 4A and Table S2). The relative abundance of Ascomycota was significantly greater in Florigraze cultivar soils (11.36 ± 0.92%) than in the soils of Arblick (9.18 ± 0.93), Ecoturf (9.27 ± 0.85) and UF_Peace cultivars (10.00 ± 0.68) (*p* < 0.05) (Table S2). Ecoturf cultivar soils had greater relative abundance of Basidiomycota (3.92 ± 0.46) than the soils of Arblick (1.51 ± 0.34), Florigraze (1.51 ± 0.44), Latitude (2.04 ± 0.54), UF_Peace (2.24 ± 0.71), and UF_Tito cultivars (1.51 ± 0.67) (*p* < 0.05) (Table S2). The relative abundance of Mortierellomycota was significantly greater in the soils of UF_Peace cultivar (3.53 ± 1.0) than in the soils of Arblick (2.56 ± 0.78) and Florigraze cultivars (1.65 ± 0.54) (*p* < 0.05) (Table S2).

**Fig. 5:**
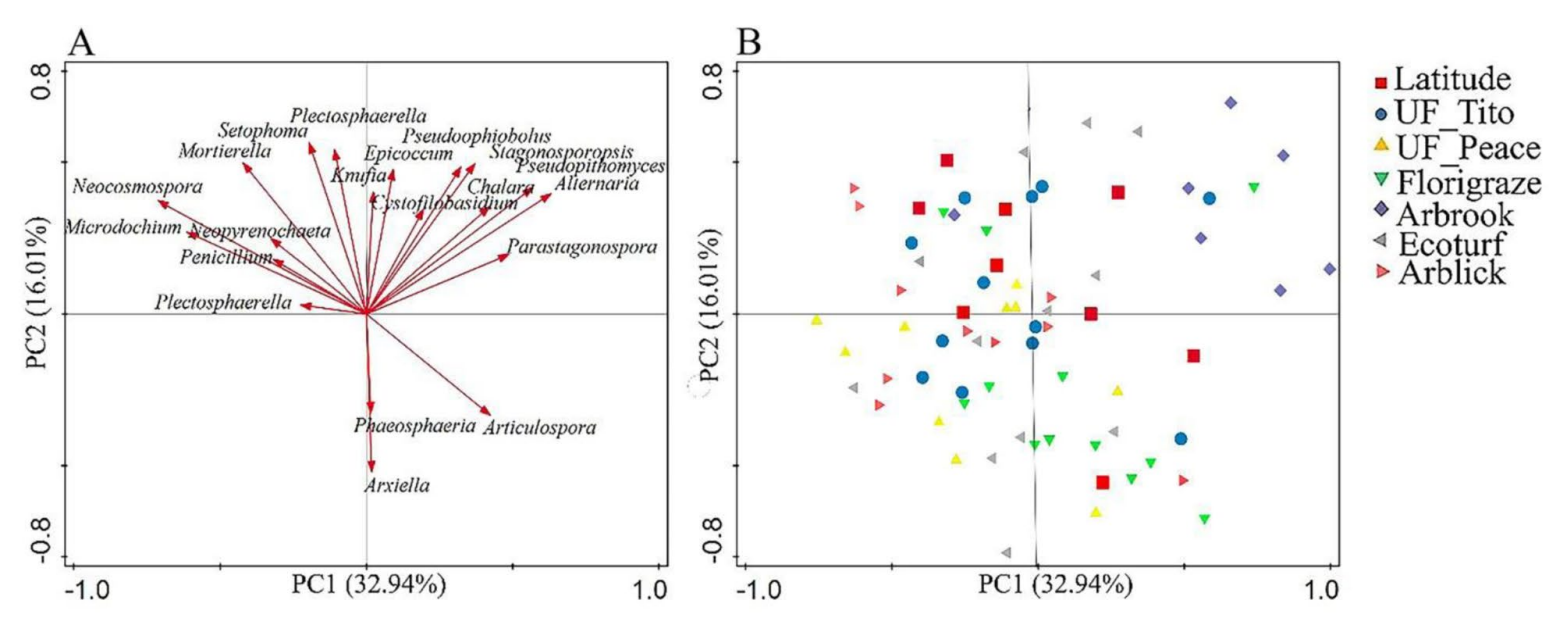
Principal component analysis of fungal (**A** and **B**) communities across RP cultivars Shapes with different colors represent rhizoma peanut cultivars (Variables) in fungi. We used taxonomic abundances data (OTUs defined at 97% sequence similarity) from 20 bacterial and fungal dominant genera samples (average abundance > 10 across all soil samples) as quantitative variables which were used to perform the PCA. The percentage of variability explained by two dimensions was given: 32.94% for the first axis and 16.01% for the second axis in fungi

The relative abundance of the dominant genera was also significantly different across RP cultivars (*p <* 0.05) (Table S3). The relative abundance of *Neocosmospora* was greater in the soils of UF_Tito cultivar (48.96 ± 9.07) than in the soils of other cultivars (*p* < 0.0001) (Fig. 4B and Table S3). The relative abundance of *Epicoccum* (21.93 ± 3.28), *Alternaria* (23.55 ± 2.21), *Stagonosporopsis* (17.44 ± 1.52), *Pseudopithomyces* (27.94 ± 3.34), *Neopyrenochaeta* (21.5 ± 2.17), and *Pleurotus* (22.77 ± 4.20) was significantly greater in the soils of Arbrook cultivar than in the soils of other cultivars (*p* < 0.0001) (Fig. 4B and Table S3). Principal component analysis results indicate the distributions of the dominant genera of fungal communities among RP cultivars (Fig. 5). The first and second principal component explains 32.94% and 16.01% of the variation, respectively. The relative abundance of the major taxonomic groups varied across different cultivars (Fig. 5, Table S3). The major taxonomic groups in the soils of UF_Tito cultivar were *Neocosmospora*, *Phaeosphaeria*, and *Microdochium*, while *Arxiella* and *Parastagonospora* were mainly present in the soils of Florigraze cultivar. *Epicoccum*, *Alternaria*, *Stagonosporopsis*, and *Pseudophiobolus* were dominant in the soils of Arbrook cultivar. The major taxonomic groups in the soils of Arblick cultivar were *Neocosmospora*, *Plectosphaerella*, and *Mortierella*, while the soils of Latitude cultivar had mainly *Neopyrenochaeta* and *Alternaria*.

### Co-occurrence networks

**Fig. 6:**
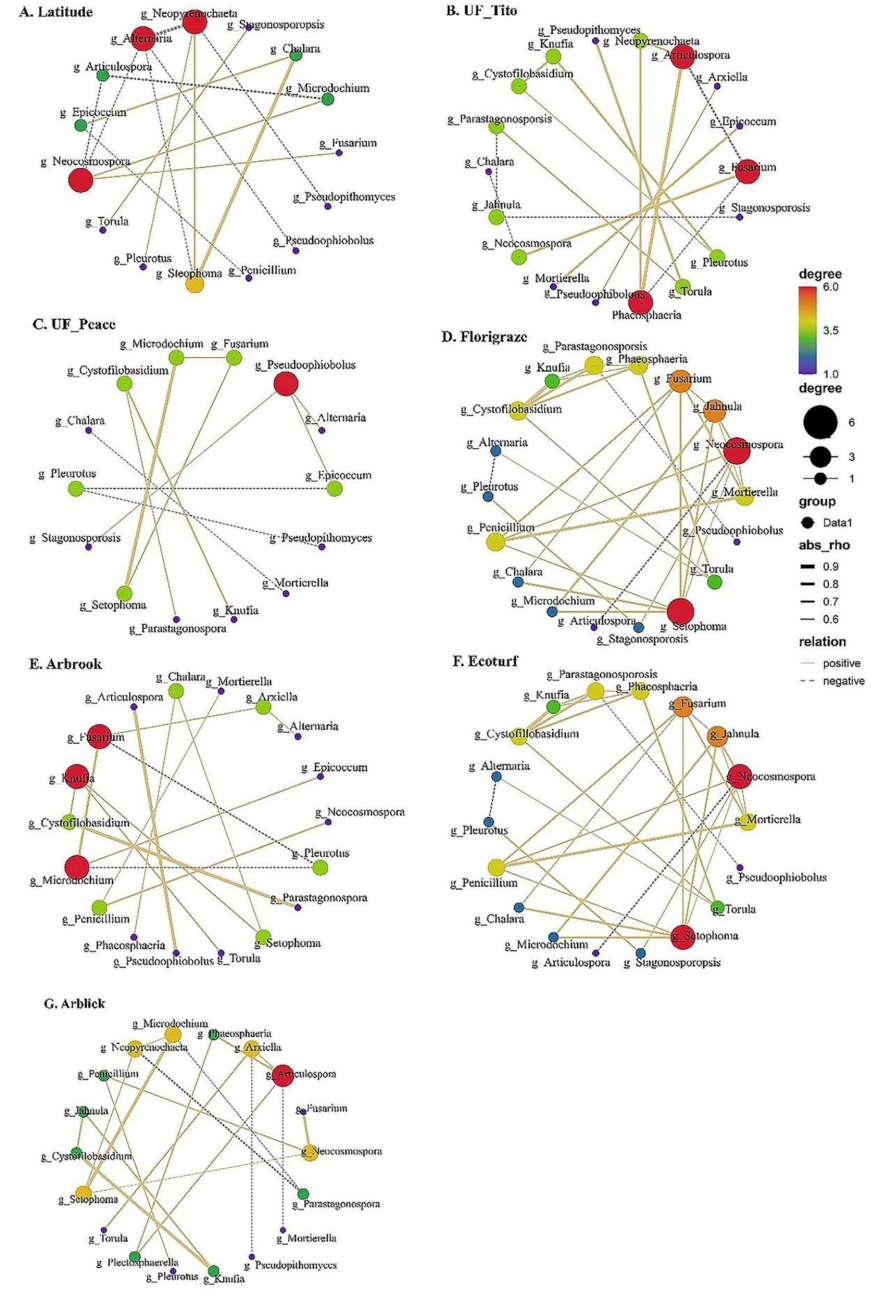
Co-occurrence network analysis of 20 dominant fungal genera among seven RP cultivars, (**A**) Latitude, (**B**) UF_Tito, (**C**) UF_Peace, (**D**) Florigraze, (**E**) Arbrook, (**F**) Ecoturf (**G**) Arblick, respectively. Each node was labelled at the genera level. A connection stand for a strong (Spearman’s *p* > 0.6) and significant (*p* < 0.05) correlation. The size of each node is in proportion to the relative abundance; the thickness of each connection between two nodes is in proportion to the value of spearman’s correlation co-efficient. Solid and dashed lines indicate positive and negative correlations, respectively

The co-occurrence patterns of the dominant fungal genera among the different RP cultivars were presented based on strong and significant taxonomic correlations. The number of positive correlations was greater than the negative correlations across seven RP cultivars (Fig. 6). There were significant differences in the co-occurrence network among RP cultivars (*p* < 0.05) (Fig. 6 and Table S4).

The average connectivity was greater in the Florigraze and Ecoturf networks, with ≥ 6 node size ranging from 15 to 20 in two networks than in other cultivar networks (*p* < 0.05) (Table S4). The keystone taxa were different in different cultivars, with *Alternaria*, *Neopyrenochaeta*, and *Neocosmospora* detected in Latitude cultivar, *Articulospora*, *Fusarium*, and *Phaeosphaeria* in UF_Tito cultivar, *Pseudophiobolus* in UF_Peace cultivar, *Neocosmospora* and *Stophoma* in Florigraze cultivar, *Fusarium*, *Knufia*, and *Microdocium* in Arbrook cultivar, *Knufia*, *Arxiella*, *Stagonosporsis*, and *Setophoma* in Ecoturf cultivar, and *Articulospora* in Arblick cultivar (Fig. 6 and Table S4).

### Functional characteristics

**Fig. 7:**
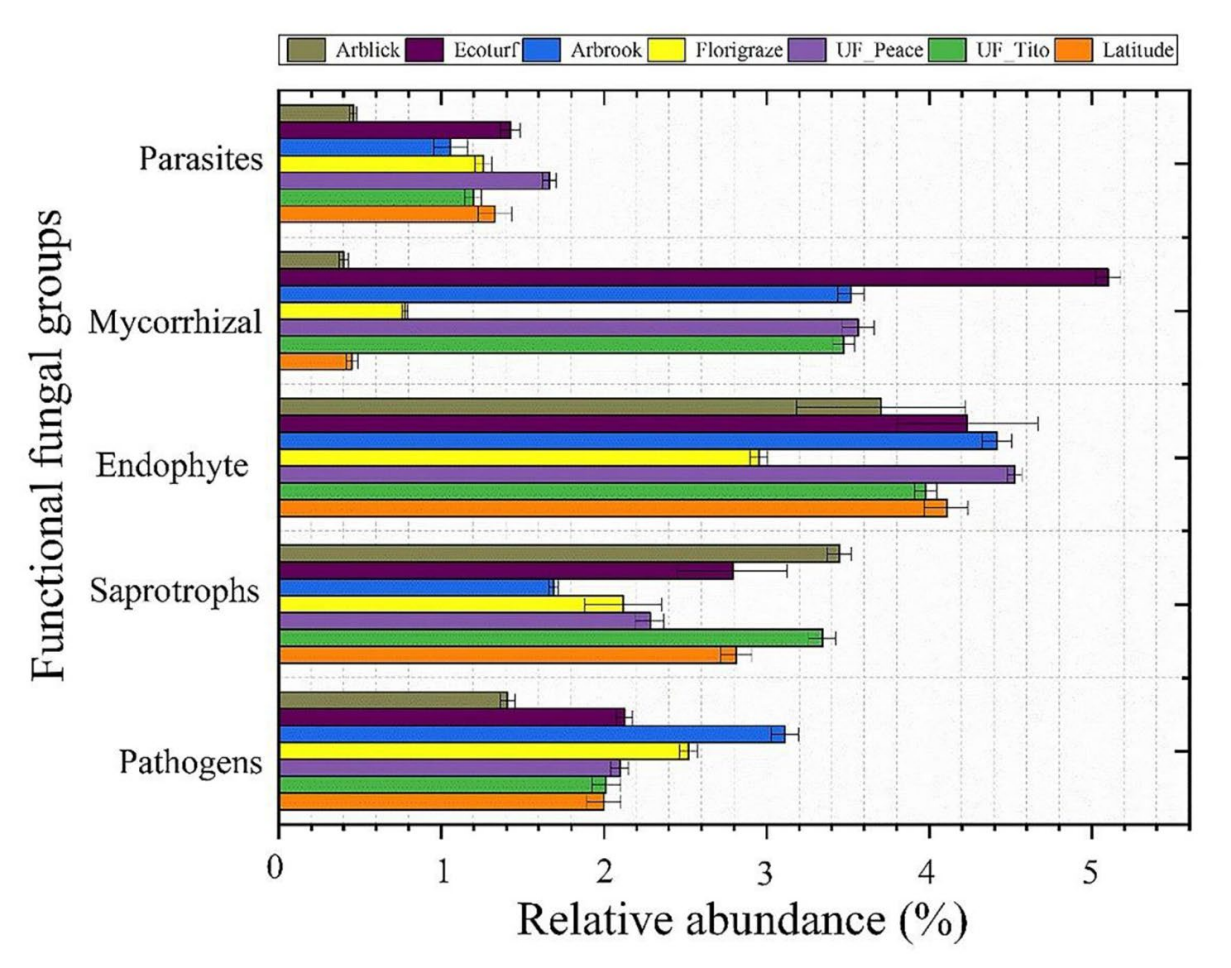
Predicted functional groups of fungal communities across cultivars. The functional groups of fungi were inferred using FUNGuild

A total of 1,853 OTUs were assigned and annotated using the FUNGuild database. The relative abundance of functional groups including pathogenic, saprotrophic, endophytic, mycorrhizal, and parasitic fungi significantly differed among RP cultivars (*p* < 0.001) (Table S5). Soils of Ecoturf cultivar had the greatest relative abundance of mycorrhizal fungal group (5.10 ± 0.44) than other cultivars (*p* < 0.0001) (Fig. 7 and Table S5). The relative abundance of the endophytic (4.52 ± 0.56) and parasitic fungi (1.67 ± 0.30) was greatest in the soils of UF_Peace cultivar than others (Fig. 7 and Table S5). There were significantly lower proportions of mycorrhizal (0.39 ± 0.08), pathogenic (1.40 ± 0.21), and parasitic fungi (0.46 ± 0.09) but higher saprotrophic fungi (3.45 ± 0.35) in the soils of Arblick cultivars than other cultivars (Fig. 7 and Table S5) (*p* < 0.0001).

## Discussion

Understanding the impact of legume forage on soil fungal communities is an important step toward identifying the overall benefits of incorporating legumes into grassland systems. Soil fungal communities play key roles in making plant nutrients available, facilitating soil nutrient cycling and promoting beneficial ecosystem services in many agricultural systems. Previous studies have shown that legume cultivars play a key role in shaping soil microbial communities [53, 54]. Our results demonstrated that RP cultivars were important determinants of soil fungal community structure and function. The findings showed that under the same soil type and conditions, different RP cultivars induced a shift in soil fungal communities. This is in line with previous reports showing that different cultivars of potato (*Solanum tuberosum* L.) and maize had significantly shifted soil fungal diversity and composition [55, 56]. For instance, Loit et al. [55] found that among twenty-one potato cultivars, Viviana, Solist, Glorieta, and Concordia significantly affect the overall fungal, pathogen, and saprotrophic community composition. Similarly, Li et al. [56] reported that cultivar Tiannuozao 60 (N) showed significant differences in fungal diversity compared with Junlong1217 (QZ) and Fujitai519 (ZL) cultivars.

Generally, patterns of fungal diversity and composition can be mediated by multiple factors including plant species and biomass, soil biotic and abiotic factors, and climatic factors [57, 58]. However, fine-scale effects of host plants on soil microbial community are often cultivar or genotype dependent. In this study, our results showed that the alpha and beta diversity of fungal communities significantly differed across RP cultivars. Ecoturf cultivar had the greatest alpha diversity than other RP cultivars. This difference might be due to the specific cultivar differences in the morphology and biochemistry of RP above and belowground components [40, 42]. In a concurrent study, Dubeux et al. [40] found out that RP cultivars differed in aboveground and belowground characteristics and BNF capacities. Ecoturf and Latitude had the greatest root + rhizome mass and N pool than Florigraze and some other cultivars. Belowground biomass serves as the main soil organic input, providing substrate for soil microorganisms, particularly soil fungi, to mediate multiple processes in soils [59].

Consistent with our study, Erhunmwunse et al. [60] identified Ascomycota, Mortierellomycota, and Basidiomycota as predominant phyla within RP systems in Florida. At the genus level, our study found that some fungal genera such as *Neocosmospora* and *Epicoccum* were dominant across all RP cultivars, suggesting their potential as key fungal indicators for RP irrespective of cultivar type. *Neocosmospora*, (previously known as *Fusarium solani* species complex), was the most abundant fungal genus, representing 21% of the fungal genera across all RP cultivars. This aligns with the findings of Erhunmwunse et al. [60] who, within the same soil type and location, reported *Fusarium* (Nectriaceae) as the primary fungal genus within Ecoturf and Florigraze RP soils. This shows the importance of this fungal genus within RP systems, warranting further investigation into their ecological roles and implications for RP management strategies.

The abundance of soil fungal genera known for their roles in nutrient cycling, plant defense, and soil health differed among RP cultivars. For example, *Phaeosphaeria* and *Microdochium* were prevalent in UF_Tito, while *Arxiella* and *Parastagonospora* were mainly present in the soils of Florigraze, and *Plectosphaerella* and *Mortierella* were dominant in the soils of Arblick cultivar. These fungal groups are responsible for nutrient cycling, decomposition of plant litter, production of antibiotics, and release of plant hormones like IAA, gibberellic acid (GA), and ACC deaminase [61, 62]. The impact of plant cultivars on soil fungal community composition is influenced by various factors including soil pH, plant root exudates, and agricultural practices. Previous studies have shown the differences in the above and belowground morphology and biochemistry of different RP cultivars [40, 42], which might have impacted soil fungal communities, in part, in our study. Studies describing the quality and quantity of root exudates specific to RP are noticeably lacking, even though differences in root exudate quality and quantity have been shown to strongly influence fungal taxa among plant cultivars [63]. Such information may provide important context for understanding the differences in soil microbial communities under RP systems.

Positive and negative edges in cooccurrence network depict interactions and competition among soil microbial communities. These interactions are crucial for ecological activities and community assemblage in any system [64]. Positive interactions play a crucial role for maintaining species diversity and ecosystem functioning [65]. In our study, co-occurrence network of dominant fungal genera among the different RP cultivars showed more positive interactions (indicating mutualism) in the forage system. The positive interactions that exist between fungi-fungi may be due to their mutualistic or commensal ecological interactions [66]. Compared to other RP cultivars, soils of Florigraze and Ecoturf RP had greater number of positive interactions. Generally, more positive interactions in network imply higher degree of cooperation and symbiotic relationships among microbial taxa. More symbiotic interactions between microbial taxa are beneficial for diversity maintenance and soil nutrient acquisition [67], which might have a positive effect on plant growth and health. In contrast, more negative interactions were observed in Latitude soil, which may be due to substrate limitation [68], and consequently leading to competition among fungal taxa in this cultivar.

Soil fungal communities can be grouped into different functional groups based on their roles in agricultural systems [69]. Symbiotrophic fungi are beneficial as they form mutualistic relationships with plants to assess plant nutrients and protect plants from diseases [70]. On the other hand, parasitic fungi can have a negative impact on plant growth and development by causing diseases such as root rot, leaf spot, powdery mildew, and rust, and by redirecting plant nutrients and resources for their growth [71, 72]. Overall, the results of FUNGuild, a functional analysis of fungal communities, revealed that the relative abundance of endophytes, mycorrhizal, and saprotrophs were significantly greater among RP cultivars compared to parasites and pathogens (Fig. 6 and Table S5). This suggests that the presence of RP stimulated the relative abundance of beneficial soil fungal communities. The implication of this is that the incorporation of RP into any grassland systems may promote soil fungal community shifts that may benefit plant growth and soil nutrient cycling. Understanding how fungal communities vary across RP cultivars can provide insights into plant-microbe interactions, disease resistance, and overall crop health, with implications for healthy and sustainable pasture management strategies.

## Conclusion

This study showed that RP cultivars significantly affected fungal diversity, composition, taxonomic interactions and functions. We found that fungal alpha diversity estimated using Shannon, Simpson, and Pielou’s evenness was significantly greater in Ecoturf. Moreover, the fungal phyla Ascomycota, Mortierellomycota and Basidiomycota​​​​​​​ were found as keystone species and were dominant in Florigraze, UF_Peace, and Ecoturf, respectively. The relative abundance of *Neocosmospora* was greater in the soil of UF_Tito. In general, RP cultivars had greater relative abundances of endophytes, mycorrhizal, and saprotrophs than of parasites and pathogens. However, compared to other RP cultivars, the soils of UF_Peace, Ecoturf, and Arblick had greater abundances of saprotrophs, endophytes, and mycorrhizal functional groups. Our findings provide evidence of crop cultivar’s effect in shaping fine-scale microbial patterns in legume-based forage systems. We highlight an importance of crop-associated soil microbiome in agroecosystems and suggesting that interacting different RP cultivars with beneficial soil fungi could be a new path for improving crop productivity, soil nutrient availability, and minimizing farm input costs through applying fungal biota as biofertilizer resource for sustainable agroecosystems.

### Electronic supplementary material

Below is the link to the electronic supplementary material.

Supplementary Material 1

## Data Availability

The sequenced raw dataset generated in this study have been submitted to the National Center for Biotechnology Information (NCBI) with BioProject ID: PRJNA1099792, (https://www.ncbi.nlm.nih.gov/sra/PRJNA1099792).
