# Supplementary material for: Soil fungal community structure and function response to rhizoma perennial peanut cultivars
Source: BMC Plant Biol. 2024 Jun 19;24:582. doi: 10.1186/s12870-024-05209-y (PMC11186081; doi:10.1186/s12870-024-05209-y)
Supplement: Supplementary file 1 — Supplementary Material 1 [file 12870_2024_5209_MOESM1_ESM.docx]

**Table S1** Analysis of variance (ANOVA) for fungal alpha diversity calculated by Simpson index and Phylogenetic index (PD), Shannon index, Pielou’s evenness (mean ± SD) among seven different rhizoma peanut cultivars in April. Different letters indicate significance at 0.05 (*p*<0.05). The significance was analyzed based on the ANOVA with Tukey’s HSD test. *p* values were shown by the ANOVA test.

| **Alpha indices** | **Arblick** | **Arbrook** | **Ecoturf** | **Florigraze** | **Latitude** | **UF_Peace** | **UF_Tito** | ***Adjusted p*-value** |
| --- | --- | --- | --- | --- | --- | --- | --- | --- |
| **Simpson index** | 0.80±0.032ab | 0.81±0.040ab | 0.84±0.039a | 0.72±0.041c | 0.77±0.019b | 0.70±0.038c | 0.82±0.044a | <0.0001 |
| **PD index** | 172.07±8.15a | 144.86±9.81b | 183.31±10.17a | 179.88±7.90a | 77±6.8bc | 173.48±12.63a | 179.98±5.8a | <0.0001 |
| **Shannon index** | 2.75±0.15c | 2.84±0.11bc | 3.04±0.13a | 2.52±0.12d | 2.80±0.12bc | 2.47±0.15d | 2.95±0.10ab | <0.0001 |
| **Pielou's evenness** | 0.58±0.022c | 0.60±0.019bc | 0.64±0.025a | 0.53±0.018d | 0.59±0.033bc | 0.52±0.022d | 0.61±0.023ab | 0.0037 |

**Table S2.** Analysis of variance (ANOVA) for the relative abundances of dominant phyla (mean±SD) of fungal communities. Different letters indicate significance at 0.05 (*p*<0.05). The significance was analyzed based on the ANOVA with Tukey’s HSD test. *p* values were shown by the ANOVA test.

| Phylum | Arblick | Arbrook | Ecoturf | Florigraze | Latitude | UF_Peace | UF_Tito | % | *Adjusted*  *P* value |  |
| --- | --- | --- | --- | --- | --- | --- | --- | --- | --- | --- |
| Ascomycota  Basidiomycota  Mortierellomycota  Chytridiomycota  Rozellomycota  Olpidiomycota  Calcarisporiellomycota  Glomeromycota | 9.18±0.93c  1.51±0.34b  2.56±0.78bc  0.67±0.38a  0.36±0.10ab  0.45±0.26b  0.27±0.13ab  0.15±0.08ab | 10.19±1.01abc  3.40±1.05a  3.09±0.55ab  0.28±0.09bc  0.12±0.03b  0.11±0.01d  0.12±0.10abc  0.04±0.02b | 9.27±0.85c  3.92±0.46a  3.35±0.62ab  0.20±0.05bc  0.75±0.59a  0.27±0.08bcd  0.09±0.03bc  0.05±0.02ab | 11.36±0.92a  1.51±0.44b  1.65±0.54c  0.08±0.02c  0.31±0.16ab  0.125±0.03cd  0.21±0.08abc  0.2±0.06ab | 10.34±0.30ab  2.04±0.54b  2.96±0.42ab  0.65±0.31a  0.27±0.07ab  0.51±0.27ab  0.3±0.13a  0.16±0.09ab | 10±0.68bc  2.24±0.71b  3.53±1.0a  0.22±0.11bc  0.33±0.12ab  0.78±0.30a  0.08±0.02bc  0.05±0.02b | 10.55±0.27ab  1.51±0.67b  3.27±0.80ab  0.40±0.24ab  0.39±0.18ab  0.36±0.14bc  0.06±0.02c  0.34±0.27a | 13.34  2.99  3.82  0.46  0.49  0.48  0.21  0.19 | <0.0001  <0.0001  <0.0001  <0.0001  0.016  <0.0001  <0.0001  0.027 |  |

**Table S3**. Analysis of variance (ANOVA) for the relative abundances of dominant genera (mean±SD) of fungal communities. Different letters indicate significance at 0.05 (*p*<0.05). The significance was analyzed based on the ANOVA with Tukey’s HSD test. *p* values were shown by the ANOVA test.

|  | Arblick | Arbrook | Ecoturf | Florigraze | Latitude | UF_Peace | UF_Tito % |  | Adjusted *P* value |
| --- | --- | --- | --- | --- | --- | --- | --- | --- | --- |
| Neocosmospora  Epicoccum  Arxiella  Mortierella  Phaeosphaeria  Alternaria  Articulospora  Stagonosporopsis  Pseudopithomyces  Neopyrenochaeta  Penicillium  Pleurotus  Pseudoophiombolus  Plectosphaerella  Fusarium  Torula  Chalara  Microdochium  phoma  Parastagonospora  Knufia  Cystofilobasidium  Jahnula | 40.72±5.46a  7.56±1.87d  5.06±0.74b  4.16±0.46a  7.75±1.58a  1.90±1.02c  2.80±1.12c  4.36±1.20b  5.27±1.08b  5.5±1.5c  4.5±1.24ab  1.18±0.60d  0.63±0.17d  7.40±2.17a  1.56±0.41a  1.15±0.30ab  0.45±0.26c  2.72±0.78b  1.77±0.98b  0.81±0.75b  1.37±0.67bc  1.88±0.77ab  2.54±0.82a | 12.18±1.98e  21.93±3.28a  3.33±0.62c  3.34±0.44b  3.39±0.57c  23.55±2.21a  6.51±1.79a  17.44±1.52a  27.94±3.34a  21.5±2.17a  4.11±1.24ab  22.77±4.20a  8.11±1.13a  1.55±0.72bc  0.38±0.22a  1.08±0.46b  3.22±0.62ab  0.88±0.54c  1.88±0.54b  1.38±0.78b  1.24±0.49bc  0.91±0.41bc  0.21±0.10d | 34.78±9.23bc  11.85±2.82c  5.84±1.83b  3.56±0.67ab  7.81±1.31a  5±2.01bc  5.43±1.19a  6.90±3.65b  5.54±1.72b  5.63±1.35c  2.5±0.80c  3.90±1.72cd  2.95±1.80c  2.72±1.12b  1.18±0.35a  1.65±0.56a  2.36±1.02b  1.86±1bc  1.90±0.76b  0.54±0.18b  1.13±0.80c  0.82±0.48c  1.09±0.94bc | 14.25±2.34e  16.88±2.82b  8.13±2.11a  1.91±0.66c  5.19±1.29b  4.58±1.42bc  5.25±2.12ab  4.58±1.20b  6.5±2.1b  5.62±4.22c  5.20±0.91a  2.05±1.63cd  2.79±1.21c  0.91±0.13c  1.43±0.66a  1.02±0.40b  1.12±0.80c  1.91±1.32bc  1.62±0.64b  4.62±1.87a  2.15±0.93ab  2.38±1.10a  0.33±0.11bc | 26.13±2.2cd  9.88±1.69cd  6±1.13b  3.16±0.62b  5.4±0.84b  6.7±1.29b  3.55±0.51c  4.55±1.14b  5.95±0.68b  12.9±2.29b  3.75±0.42bc  6.6±1.5bc  4.85±0.70b  0.55±0.15c  0.8±0.33a  1.12±0.27ab  3.7±0.82a  1.1±0.73c  2.45±0.59b  1±0.70b  1.63±0.64bc  1.29±0.30bc  1.4±0.80b | 20.11±9.49de  7.41±0.65d  5.80±1.30b  3.60±0.53ab  8.13±1.15a  1.91±0.51c  3.63±0.61bc  7.12±1.15b  2.29±1.15b  5.37±1.15c  4±0.67ab  2.8±1.02cd  0.16±0.09d  1.25±0.58c  7±6.18a  0.79±0.39b  0.33±0.18c  2.91±0.99ab  1.58±1.04b  0.83±0.77b  0.95±0.62c  0.70±0.20c  0.58±0.32bc | 48.96±9.07a 21.45  12±3.78c 7.92  6±1.22b 5.26  3.66±0.52ab 2.63  8.72±2.16a 4.62  2.27±1.67bc 5.11  3.60±0.86bc 3.40  6.18±1.41b 6.23  5.95±1.27b 8.18  4.22±1.16c 7.03  5±1.18ab 3.04  9.09±2.02b 5.7  1.54±1.21cd 3.01  1.04±0.35c 2.20  1.54±0.68a 1.01  1.32±0.42ab 0.69  2.81±1.07ab 2.08  4.36±1.80a 2.29  3.55±0.90a 1.74  1.63±0.50b 1.82  2.80±0.53a 1.34  2.41±1.03a 1.43  2.81±1.25a 1.71 |  | <0.0001  <0.0001  <0.0001  <0.0001  <0.0001  <0.0001  <0.0001  <0.0001  <0.0001  <0.0001  <0.0001  <0.0001  <0.0001  <0.0001  0.047  <0.001  <0.0001  <0.0001  <0.0001  <0.0001  <0.0001  <0.0001  <0.0001 |

**Table S4** Co-occurrence network analysis of fungal-dominated genera across seven different RP cultivars. Correlation types are showing positive and negative edges associated with the source and target. *p* values were corrected by False Discovery Rate (FRD).

| **Source** | **Target** | **rho** | **Edge Width** | **Node Size** | **Adjusted p-value** | **Correlation_type** |
| --- | --- | --- | --- | --- | --- | --- |
| **Latitude** |  |  |  |  |  |  |
| g_Neocosmospora | g_Alternaria | 0.703 | 0.703 | 15 | 0.023 | positive |
| g_Neocosmospora | g_Articulospora | 0.724 | 0.724 | 15 | 0.017 | positive |
| g_Neocosmospora | g_Fusarium | 0.747 | 0.747 | 15 | 0.013 | positive |
| g_Neocosmospora | g_Microdochium | 0.712 | 0.712 | 15 | 0.020 | positive |
| g_Epicoccum | g_Penicillium | 0.688 | 0.688 | 8.3 | 0.027 | positive |
| g_Epicoccum | g_Chalara | 0.756 | 0.756 | 8.3 | 0.011 | positive |
| g_Alternaria | g_Neopyrenochaeta | 0.871 | 0.871 | 15 | 0.001 | positive |
| g_Alternaria | g_Pseudoophiobolus | -0.662 | 0.662 | 15 | 0.036 | negative |
| g_Alternaria | g_Setophoma | -0.699 | 0.699 | 15 | 0.024 | negative |
| g_Articulospora | g_Microdochium | -0.646 | 0.646 | 8.3 | 0.043 | negative |
| g_Stagonosporopsis | g_Torula | -0.849 | 0.849 | 5 | 0.001 | negative |
| g_Pseudopithomyces | g_Neopyrenochaeta | -0.666 | 0.666 | 5 | 0.035 | negative |
| g_Neopyrenochaeta | g_Pleurotus | -0.647 | 0.647 | 15 | 0.043 | negative |
| g_Neopyrenochaeta | g_Setophoma | -0.768 | 0.768 | 15 | 0.009 | negative |
| g_Chalara | g_Setophoma | -0.689 | 0.689 | 8.3 | 0.027 | negative |
| **UF_Tito** |  |  |  |  |  |  |
| g_Neocosmospora | g_Fusarium | 0.821 | 0.821 | 12.5 | 0.003 | positive |
| g_Neocosmospora | g_Chalara | 0.793 | 0.793 | 12.5 | 0.006 | positive |
| g_Epicoccum | g_Mortierella | 0.707 | 0.707 | 5 | 0.022 | positive |
| g_Arxiella | g_Pseudoophiobolus | 0.905 | 0.905 | 5 | <0.001 | positive |
| g_Phaeosphaeria | g_Articulospora | 0.812 | 0.812 | 20 | 0.004 | positive |
| g_Phaeosphaeria | g_Neopyrenochaeta | 0.733 | 0.733 | 20 | 0.015 | positive |
| g_Phaeosphaeria | g_Fusarium | 0.825 | 0.825 | 20 | 0.003 | positive |
| g_Articulospora | g_Neopyrenochaeta | 0.793 | 0.793 | 20 | 0.006 | positive |
| g_Articulospora | g_Fusarium | 0.644 | 0.644 | 20 | 0.044 | positive |
| g_Stagonosporopsis | g_Jahnula | 0.728 | 0.728 | 5 | 0.016 | positive |
| g_Pseudopithomyces | g_Torula | 0.792 | 0.792 | 5 | 0.006 | positive |
| g_Pleurotus | g_Knufia | -0.673 | 0.673 | 12.5 | 0.032 | negative |
| g_Pleurotus | g_Cystofilobasidium | -0.680 | 0.680 | 12.5 | 0.030 | negative |
| g_Torula | g_Parastagonospora | -0.795 | 0.795 | 12.5 | 0.005 | negative |
| g_Parastagonospora | g_Jahnula | -0.688 | 0.688 | 12.5 | 0.027 | negative |
| g_Knufia | g_Cystofilobasidium | -0.701 | 0.701 | 12.5 | 0.023 | negative |
| **UF_Peace** |  |  |  |  |  |  |
| g_Epicoccum | g_Pleurotus | 0.650 | 0.650 | 12.5 | 0.022 | positive |
| g_Epicoccum | g_Pseudoophiobolus | 0.598 | 0.598 | 12.5 | 0.039 | positive |
| g_Mortierella | g_Chalara | 0.582 | 0.582 | 5 | 0.046 | positive |
| g_Alternaria | g_Pseudoophiobolus | 0.722 | 0.722 | 5 | 0.0079 | positive |
| g_Stagonosporopsis | g_Pseudoophiobolus | 0.722 | 0.722 | 5 | 0.0079 | positive |
| g_Pseudopithomyces | g_Pleurotus | 0.983 | 0.983 | 20 | <0.0001 | positive |
| g_Fusarium | g_Microdochium | 0.656 | 0.656 | 12.5 | 0.020 | positive |
| g_Fusarium | g_Setophoma | 0.764 | 0.764 | 12.5 | 0.003 | positive |
| g_Microdochium | g_Setophoma | -0.711 | 0.711 | 12.5 | 0.009 | negative |
| g_Parastagonospora | g_Cystofilobasidium | -0.582 | 0.582 | 5 | 0.046 | negative |
| g_Knufia | g_Cystofilobasidium | -0.618 | 0.618 | 5 | 0.032 | negative |
| **Florigraze** |  |  |  |  |  |  |
| g_Neocosmospora | g_Mortierella | 0.640 | 0.640 | 20 | 0.024 | positive |
| g_Neocosmospora | g_Articulospora | 0.632 | 0.632 | 20 | 0.027 | positive |
| g_Neocosmospora | g_Stagonosporopsis | 0.694 | 0.694 | 20 | 0.012 | positive |
| g_Neocosmospora | g_Penicillium | 0.597 | 0.597 | 20 | 0.040 | positive |
| g_Neocosmospora | g_Setophoma | 0.714 | 0.714 | 20 | 0.008 | positive |
| g_Neocosmospora | g_Jahnula | 0.859 | 0.859 | 20 | <0.001 | positive |
| g_Mortierella | g_Penicillium | 0.747 | 0.747 | 14 | 0.005 | positive |
| g_Mortierella | g_Fusarium | 0.593 | 0.593 | 14 | 0.041 | positive |
| g_Mortierella | g_Jahnula | 0.766 | 0.766 | 14 | 0.003 | positive |
| g_Phaeosphaeria | g_Torula | 0.600 | 0.600 | 14 | 0.039 | positive |
| g_Phaeosphaeria | g_Parastagonospora | 0.644 | 0.644 | 14 | 0.023 | positive |
| g_Phaeosphaeria | g_Knufia | 0.776 | 0.776 | 14 | 0.003 | positive |
| g_Phaeosphaeria | g_Cystofilobasidium | 0.579 | 0.579 | 14 | 0.048 | positive |
| g_Alternaria | g_Pleurotus | 0.715 | 0.715 | 8 | 0.008 | positive |
| g_Alternaria | g_Torula | 0.750 | 0.750 | 8 | 0.004 | positive |
| g_Stagonosporopsis | g_Pleurotus | 0.657 | 0.657 | 8 | 0.020 | positive |
| g_Penicillium | g_Fusarium | 0.605 | 0.605 | 14 | 0.037 | positive |
| g_Penicillium | g_Setophoma | 0.752 | 0.752 | 14 | 0.004 | positive |
| g_Pseudoophiobolus | g_Parastagonospora | 0.599 | 0.599 | 5 | 0.039 | positive |
| g_Fusarium | g_Chalara | 0.650 | 0.650 | 17 | 0.021 | positive |
| g_Fusarium | g_Setophoma | 0.795 | 0.795 | 17 | 0.001 | positive |
| g_Fusarium | g_Jahnula | 0.792 | 0.792 | 17 | 0.002 | positive |
| g_Torula | g_Cystofilobasidium | 0.773 | 0.773 | 11 | 0.003 | positive |
| g_Chalara | g_Setophoma | 0.757 | 0.757 | 8 | 0.004 | positive |
| g_Microdochium | g_Setophoma | 0.649 | 0.649 | 8 | 0.022 | positive |
| g_Microdochium | g_Jahnula | 0.814 | 0.814 | 8 | 0.001 | positive |
| g_Setophoma | g_Jahnula | 0.945 | 0.945 | 20 | <0.0001 | positive |
| g_Parastagonospora | g_Knufia | -0.720 | 0.720 | 14 | 0.0082 | negative |
| g_Parastagonospora | g_Cystofilobasidium | -0.704 | 0.704 | 14 | 0.010 | negative |
| g_Knufia | g_Cystofilobasidium | -0.577 | 0.577 | 11 | 0.049 | negative |
| **Arbrook** |  |  |  |  |  |  |
| g_Neocosmospora | g_Penicillium | 0.811 | 0.811 | 5 | 0.007 | positive |
| g_Epicoccum | g_Microdochium | 0.762 | 0.762 | 5 | 0.016 | positive |
| g_Arxiella | g_Alternaria | 0.680 | 0.680 | 12.5 | 0.043 | positive |
| g_Arxiella | g_Fusarium | 0.730 | 0.730 | 12.5 | 0.025 | positive |
| g_Mortierella | g_Penicillium | 0.686 | 0.686 | 5 | 0.041 | positive |
| g_Phaeosphaeria | g_Chalara | 0.718 | 0.718 | 5 | 0.029 | positive |
| g_Articulospora | g_Pseudoophiobolus | 0.957 | 0.957 | 5 | <0.0001 | positive |
| g_Pleurotus | g_Fusarium | 0.862 | 0.862 | 12.5 | 0.002 | positive |
| g_Pleurotus | g_Microdochium | 0.754 | 0.754 | 12.5 | 0.018 | positive |
| g_Fusarium | g_Microdochium | 0.709 | 0.709 | 20 | 0.032 | positive |
| g_Torula | g_Knufia | 0.744 | 0.744 | 5 | 0.021 | positive |
| g_Chalara | g_Setophoma | 0.970 | 0.970 | 12.5 | <0.0001 | positive |
| g_Setophoma | g_Knufia | 0.794 | 0.794 | 12.5 | 0.0104 | positive |
| g_Parastagonospora | g_Cystofilobasidium | -0.784 | 0.784 | 5 | 0.0123 | negative |
| g_Knufia | g_Cystofilobasidium | -0.701 | 0.701 | 20 | 0.0352 | negative |
| **Ecoturf** |  |  |  |  |  |  |
| g_Epicoccum | g_Arxiella | 0.669 | 0.669 | 10 | 0.024 | positive |
| g_Epicoccum | g_Alternaria | 0.624 | 0.624 | 10 | 0.039 | positive |
| g_Arxiella | g_Phaeosphaeria | 0.634 | 0.634 | 20 | 0.036 | positive |
| g_Arxiella | g_Knufia | 0.655 | 0.655 | 20 | 0.028 | positive |
| g_Arxiella | g_Cystofilobasidium | 0.731 | 0.731 | 20 | 0.01 | positive |
| g_Mortierella | g_Phaeosphaeria | 0.647 | 0.647 | 15 | 0.031 | positive |
| g_Mortierella | g_Setophoma | 0.685 | 0.685 | 15 | 0.019 | positive |
| g_Mortierella | g_Knufia | 0.662 | 0.662 | 15 | 0.026 | positive |
| g_Alternaria | g_Pseudopithomyces | 0.643 | 0.643 | 10 | 0.032 | positive |
| g_Stagonosporopsis | g_Pseudopithomyces | 0.681 | 0.681 | 20 | 0.020 | positive |
| g_Stagonosporopsis | g_Chalara | 0.722 | 0.722 | 20 | 0.012 | positive |
| g_Stagonosporopsis | g_Parastagonospora | 0.609 | 0.609 | 20 | 0.046 | positive |
| g_Stagonosporopsis | g_Cystofilobasidium | 0.821 | 0.821 | 20 | 0.001 | positive |
| g_Pseudopithomyces | g_Parastagonospora | 0.637 | 0.637 | 15 | 0.034 | positive |
| g_Pleurotus | g_Parastagonospora | 0.851 | 0.851 | 5 | 0.0008 | positive |
| g_Plectosphaerella | g_Setophoma | 0.924 | 0.924 | 10 | <0.0001 | positive |
| g_Chalara | g_Setophoma | -0.688 | 0.688 | 15 | 0.019 | negative |
| g_Chalara | g_Knufia | -0.804 | 0.804 | 15 | 0.002 | negative |
| g_Microdochium | g_Setophoma | -0.826 | 0.826 | 5 | 0.001 | negative |
| g_Knufia | g_Cystofilobasidium | -0.847 | 0.847 | 20 | 0.0009 | negative |
| **Arblick** |  |  |  |  |  |  |
| g_Neocosmospora | g_Penicillium | 0.716 | 0.716 | 15 | 0.013 | positive |
| g_Neocosmospora | g_Fusarium | 0.897 | 0.897 | 15 | 0.0001 | positive |
| g_Neocosmospora | g_Setophoma | 0.603 | 0.603 | 15 | 0.049 | positive |
| g_Arxiella | g_Articulospora | 0.612 | 0.612 | 15 | 0.044 | positive |
| g_Arxiella | g_Pseudopithomyces | 0.734 | 0.734 | 15 | 0.009 | positive |
| g_Arxiella | g_Torula | 0.830 | 0.830 | 15 | 0.001 | positive |
| g_Mortierella | g_Articulospora | 0.712 | 0.712 | 5 | 0.013 | positive |
| g_Phaeosphaeria | g_Articulospora | 0.693 | 0.693 | 10 | 0.017 | positive |
| g_Phaeosphaeria | g_Plectosphaerella | 0.634 | 0.634 | 10 | 0.035 | positive |
| g_Articulospora | g_Plectosphaerella | 0.655 | 0.655 | 20 | 0.028 | positive |
| g_Neopyrenochaeta | g_Microdochium | 0.681 | 0.681 | 15 | 0.02 | positive |
| g_Neopyrenochaeta | g_Setophoma | 0.973 | 0.973 | 15 | <0.0001 | positive |
| g_Neopyrenochaeta | g_Parastagonospora | 0.788 | 0.788 | 15 | 0.003 | positive |
| g_Penicillium | g_Pleurotus | 0.786 | 0.786 | 10 | 0.004 | positive |
| g_Microdochium | g_Setophoma | 0.947 | 0.947 | 15 | <0.0001 | positive |
| g_Microdochium | g_Parastagonospora | -0.634 | 0.634 | 15 | 0.035 | negative |
| g_Jahnula | g_Knufia | -0.625 | 0.625 | 10 | 0.039 | negative |
| g_Jahnula | g_Cystofilobasidium | -0.808 | 0.808 | 10 | 0.002 | negative |
| g_Knufia | g_Cystofilobasidium | -0.655 | 0.655 | 10 | 0.028 | negative |

**Table S5** Analysis of variance (ANOVA) for fungal functional groups (mean ± SD) among seven different rhizoma peanut cultivars in April. Different letters indicate significance at 0.05 (*p*<0.05). The significance was analyzed based on the ANOVA with Tukey’s HSD test. *p* values were shown by the ANOVA test.

| **Functional Groups** | **Latitude** | **UF_Tito** | **UF_Peace** | **Florigraze** | **Arbrook** | **Ecoturf** | **Arblick** | **Adjusted**  ***p*-value** |
| --- | --- | --- | --- | --- | --- | --- | --- | --- |
| Pathogens | 2±0.29c | 2.01±0.22b | 2.10±0.37b | 2.52±0.43b | 3.08±0.21a | 2.12±0.30b | 1.40±0.21b | 0.0008 |
| Saprotrophs | 2.81±0.35ab | 3.34±0.23ab | 2.27±0.38c | 2.12±0.23b | 1.69±0.35c | 2.79±0.40b | 3.45±0.35a | <0.0001 |
| Endophyte | 4.10±0.35b | 3.97±0.37b | 4.52±0.56d | 2.96±0.36bc | 4.41±0.53a | 4.23±0.43b | 3.70±0.52cd | <0.0001 |
| Mycorrhizal | 0.45±0.046c | 3.47±0.46b | 3.56±0.33b | 0.77±0.084c | 3.51±0.61b | 5.10±0.44a | 0.39±0.088c | <0.0001 |
| Parasites | 1.33±0.34bc | 1.20±0.39cd | 1.67±0.30a | 1.26±0.30bcd | 1.05±0.33d | 1.42±0.21b | 0.46±0.093e | <0.0001 |
